# Travel burdens to access care among children with cancer between 2016 and 2019: Analysis of a national population-based cancer registry in Japan

**DOI:** 10.1371/journal.pone.0300840

**Published:** 2024-04-16

**Authors:** Anna Tsutsui, Yoshitaka Murakami, Satomi Okamura, Takako Fujimaki, Masayuki Endo, Yuko Ohno

**Affiliations:** 1 Department of Medical Statistics, School of Medicine, Toho University, Tokyo, Japan; 2 Department of Mathematical Health Science, Graduate School of Medicine, Osaka University, Suita, Osaka, Japan; 3 Department of Medical Innovation, Osaka University Hospital, Suita, Osaka, Japan; 4 Division of Information Science, Graduate School of Science and Technology, Nara Institute of Science and Technology, Nara, Japan; 5 Department of Obstetrics and Gynecology, Graduate School of Medicine, Osaka University, Suita, Osaka, Japan; 6 Department of Children’s and Women’s Health, Division of Health Science, Graduate School of Medicine, Osaka University, Suita, Osaka, Japan; 7 Department of Mathematical Science, Graduate School of Engineering Science, Osaka University, Suita, Osaka, Japan; London School of Hygiene & Tropical Medicine Centre of Global Change and Health: London School of Hygiene & Tropical Medicine, UNITED KINGDOM

## Abstract

**Background:**

Centralization of cancer care increases survival but increases the travel burden (i.e., travel durations, distances, and expenditures) in visiting hospitals. This study investigated the travel burdens to access cancer care for children aged 18 years and younger in Japan.

**Methods:**

The study population comprised 10,709 patients diagnosed between 2016 and 2019 obtained from a national population-based cancer registry in Japan. Their residences were classified as urban or rural. We counted the number of patients treated at specialized hospitals and investigated the treatment centralization across diagnostic groups by Pareto plot. Travel burdens to access care were estimated using a route-planner web service and summarized using median values. A multivariable logistic model was performed to investigate factors associated with the events of car travel duration exceeding 1 h.

**Results:**

Of the patients, 76.7% lived in urban areas, and 82.5% received treatment in designated hospitals for childhood cancer. The Pareto plot suggested that the top five hospitals treated 63.5% of patients with retinoblastoma. The estimated travel burdens for all patients were 0.62 h (0.57 h in urban areas and 1.00 h in rural areas), 16.9 km, and 0.0 dollars of toll charges. Regarding travel duration, 21.7% of patients had travel exceeding 1 h, and rural areas, retinoblastoma, malignant bone tumors, and childhood cancer-hub hospitals were associated with travel duration exceeding 1 h (adjusted odds ratios of 6.93, 3.59, 1.94, and 1.91, respectively).

**Conclusions:**

Most patients were treated in specialized hospitals and the treatments for specific diseases were centralized. However, most patients were estimated to travel less than 1 h, and the travel burden tended to increase for patients in rural areas, those with specific diseases, and those going to specialized hospitals. Cancer control measures in Japan have steadily improved centralized treatment while keeping the travel burden relatively manageable.

## Introduction

An estimated 397,000 children (aged 0–14 years) worldwide were newly affected with cancer in 2015 [1]. Improved accessibility to specialized centers and up-to-date cancer care have enhanced survival rates [2, 3]. Additionally, most patients in high-income countries receive cancer care in specialized hospitals [2, 4]. Consequently, the 5-year survival rate has increased to over 80% [5–7], which has increased the number of childhood cancer survivors. Several of these survivors experience somatic and mental late effects induced by cancer treatment [8]. However, specialized cancer care is available in only a limited number of specialized hospitals.

A recent concern is the travel burden regarding treatment centralization, which means that patients travel to receive treatment at a limited number of specialized treatment facilities. The limited number of treatment facilities by the treatment centralization can increase travel distances and durations to access hospitals for patients from rural areas [9, 10], which can lead to more advanced disease at diagnosis, inappropriate treatment, a worse prognosis, and a worse quality of life [11], as well as travel-related financial burdens [12]. Although the negative effects of increased distance on survival are controversial [13], patients from rural and/or socioeconomically disadvantaged areas have higher mortality rates than those without these conditions [14]. Overall, parents express a willingness to travel wherever required for their children to receive optimal care [15]; however, they consistently report a lack of support for traveling, relocating, and returning home [16]. In Japan, specialized hospitals for childhood cancer have been established since 2013, when the second term of the Basic Plan to Promote Cancer Control Programs was developed [17]. The average travel duration from municipalities to the nearest specialized hospital for the 15 childhood cancer-hub hospitals is estimated at 1.8 h [18]. Most parents of children with cancer agreed with treatment centralization but disagreed with a travel duration of greater than 1 h each way [19]. One study targeting cancer patients of all ages reported that travel duration tended to be longer in areas with lower population densities [20]. Another study reported no remarkable increase in travel burdens since the establishment of childhood cancer-hub hospitals, but the study only focused on one prefecture in Japan, and the results cannot be generalized for the whole of Japan [21]. Therefore, research on childhood cancer treatment in Japan must focus on recent situations in travel burdens and investigate the factors related to increased travel burden, such as rural areas, based on real-world, nationwide data.

This study investigated the latest situations in travel burdens (i.e., travel durations, distances, and expenditures) among children with cancer to access hospitals for receiving treatment and determined the factors associated with high travel burdens. This study used data from the national population-based cancer registry (PBCR) from 2016 to 2019 in Japan.

## Materials and methods

### Setting and patients

This study used Japan’s national PBCR data, which will include all cancer cases. Under the law, all hospitals and parts of designated clinics in Japan must report cancer incidence information, and the cancer morbidity and death information collected is centrally managed by the Japanese government [17, 22].

**Fig 1:**
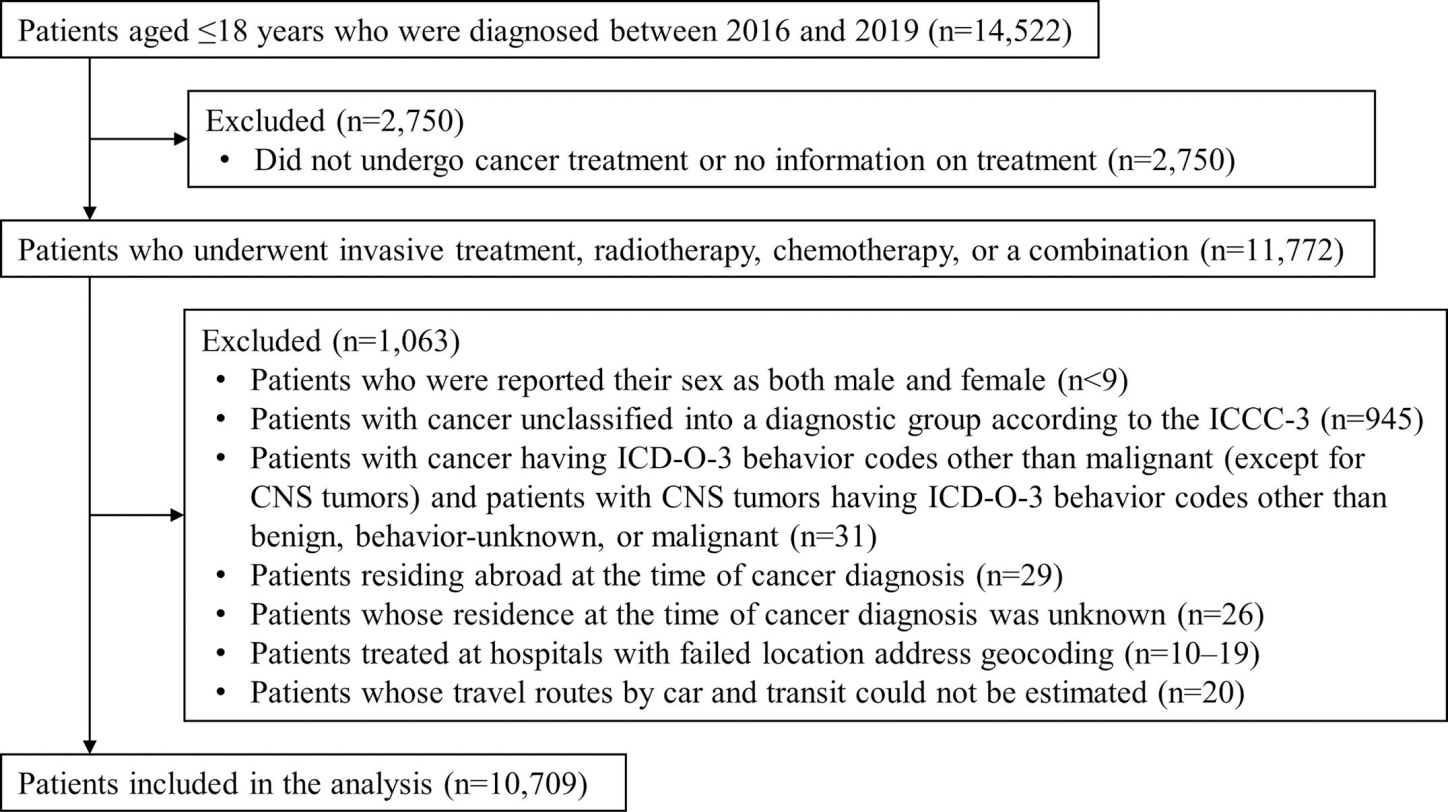
Patient flow. CNS, central nervous system; ICCC-3, 3rd edition of the International Classification of Childhood Cancer; ICD-O-3, 3rd edition of the International Classification of Diseases for Oncology.

The study population included cancer patients aged 18 years or younger and diagnosed between 2016 and 2019 (n = 14,522; Fig 1). The inclusion criteria were the patients who have received invasive treatment, radiotherapy, chemotherapy, or a combination thereof (n = 11,772). Patients with multiple cancers were included, and we analyzed information regarding the cancer with the smallest registration number, mainly assigned in ascending order of diagnosis date for each patient. The exclusion criteria were as follows: their sex reported as both male and female (n<10); cancer unclassified into a diagnostic group based on the International Classification of Childhood Cancer, third edition (n = 945) [23]; cancer whose behavior code was other than malignant (behavior code was not 3) based on the International Classification of Diseases for Oncology, third edition, though patients with central nervous system (CNS) tumors were included even if the tumor was benign (behavior code = 0) or behavior-unknown (behavior code = 1) (n = 31); living abroad (n = 29) or in an unknown location (n = 26) at the time of cancer diagnosis; treated at a hospital at the community level (e.g., Omori-nishi 5 cho-me, Ota Ward, Tokyo; Yamada-oka 2-chome, Suita City, Osaka Prefecture) that failed address geocoding (n = 10–19); and travel routes by car and public transportation (transit) that could not be estimated (n = 20). Overall, 10,709 patients were analyzed in this study.

### Estimation of travel burdens

The primary outcome measures were the minimum estimated travel duration, distance, and expenditure required for each patient’s travel from the patient’s residence to the hospitals where the patient received invasive treatment, radiotherapy, or chemotherapy. These measures were estimated by considering travel routes with the shortest travel duration by car, including the use of ferries; and by transit, including trains, airplanes, and buses. The expenditures were the sum of toll charges for a standard-sized car or the sum of transit fees for travel on the estimated route. The transit fees were for one adult and did not include the child’s fare, which varies—free, half price, or full price—depending on the child’s schooling type. The expenditures were converted from Japanese yen to U.S. dollars at 141.21 Japanese yen per U.S. dollar, based on the average central rate as of July 2023 [24].

These outcome measures regarding travel were estimated using a route planner web service [25]. To ensure privacy, digital signatures and hypertext transfer protocol secure (HTTPS) were used to facilitate secure communication over a network. Further, the following procedures were adopted to protect personal information. First, we received two datasets. One dataset included line numbers assigned sequential numbers, including many skipped numbers, and other variables required for the analysis but did not include information on the location at the community level (n = 14,522). Another dataset included only sequence numbers assigned from 1 (i.e., not line numbers) and patients’ residences and hospital locations at the community level. To ensure the patients’ privacy, this latter dataset was mixed with actual and false location data (129,910 records) before being sent to us, in accordance with the condition for obtaining data. Thereafter, we performed address geocoding to assign latitudinal and longitudinal coordinates to these location data and randomly moved all the coordinates within a 90-meter radius of the original coordinates. Thereafter, we obtained estimated travel duration, distance, and expenditure under the travel routes between each moved coordinates of the residence and hospital using a route planner web service. This was used to create a dataset that included information on travel burdens and the sequence numbers but no location information. Subsequently, we sent back the datasets to the Japanese PBCR data provider, who extracted only actual data (i.e., not taking the mixed location data) from the datasets, removed the sequence numbers, and added the line numbers. Then, we merged this with our original dataset using the line numbers as a key. Consequently, a final dataset (n = 14,522) was created for use in our study. This dataset comprised information on travel burden and patient variables but no information on patient location.

### Other variables

**Fig 2:**
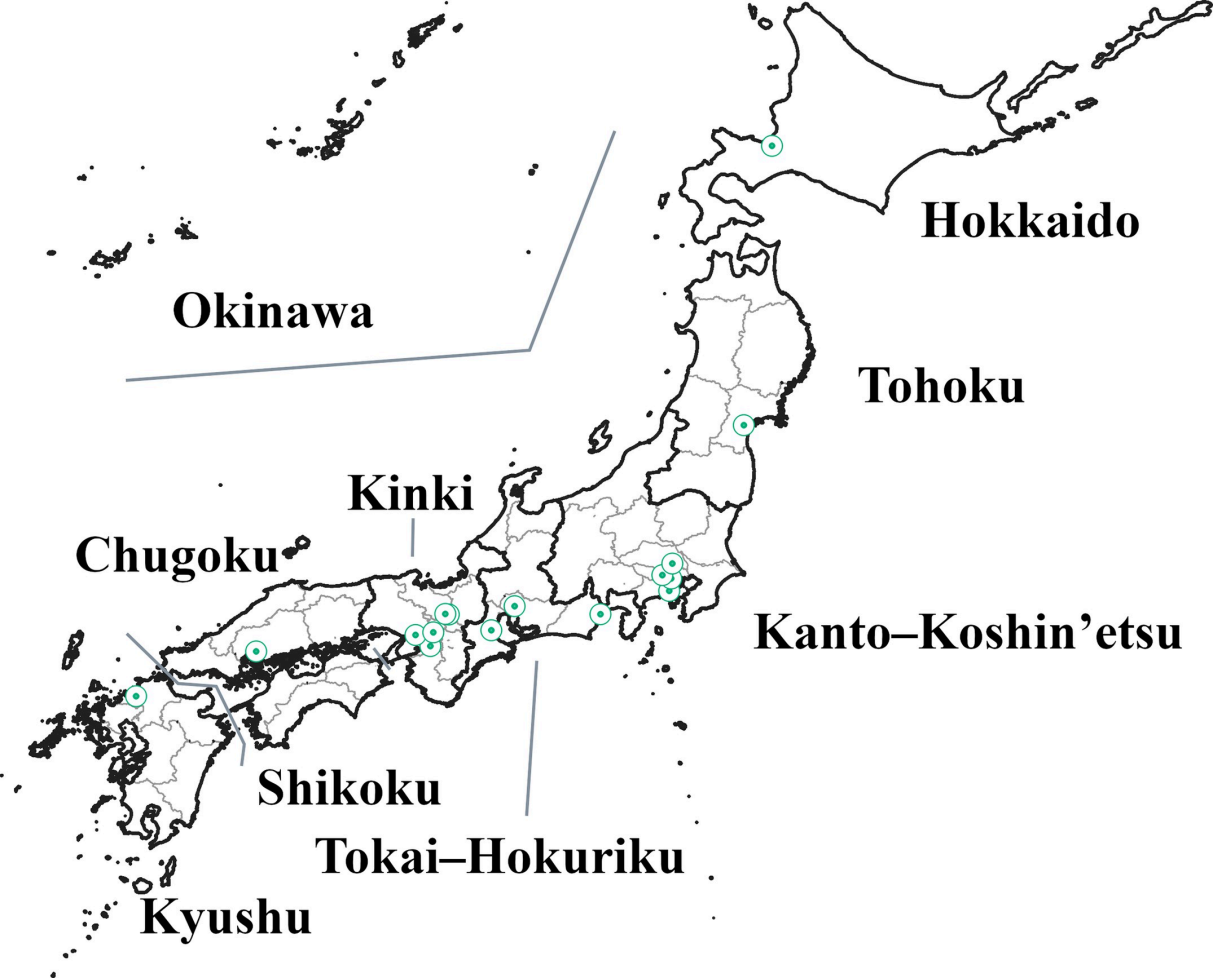
Area division and the specialized hospital in Japan. Thick lines indicate the boundaries of the seven regions. The Chugoku and Shikoku regions were combined into one region, and the Kyushu and Okinawa regions were combined into one region, resulting in seven regional divisions. Thin lines indicate the boundaries of the 47 prefectures. Green circles indicate childhood cancer-hub hospitals designated in 2013 or 2019. This map was created from data on the Digital National Land Information download service under a CC BY license, with permission from the Information Utilization Division, Real Estate and Construction Economy Bureau, Ministry of Land, Infrastructure, Transport and Tourism, original copyright 2015 and 2021, respectively [28, 29].

Additionally, we examined the combination of treatments and whether patients who received several types of treatments received all types of treatment at the same hospital. If hospital codes were different but the travel duration to each hospital was the same, the hospitals were assumed as the same. Moreover, we included the type of hospital as an outcome measure. Hospitals were classified into the following four types: (1) childhood cancer-hub hospitals designated in 2013 or 2019 (16 hospitals; Fig 2); (2) childhood cancer-specialized hospitals designated either as a central organization in 2014 or as a corporation hospital as of April 1, 2020; (3) hospitals designated in 2018 for all ages; and (4) others. Another outcome measure was whether patients received at least one treatment at a hospital in the area in which they lived, and each patient was examined according to the secondary healthcare area, prefecture, and region (Fig 2). In Japan, secondary healthcare areas refer to areas that can provide general inpatient medical care, which are zoned within each of the 47 prefectures under the Medical Care Act [26], and there are 344 such areas as of April 1, 2016. A region was classified based on the classification used for the designation of childhood cancer-hub hospitals in 2013 [27].

The grouping variable was the area of residence in urban and rural areas. Urban areas were defined as cities located in prefectural capitals or municipalities classified as urban areas in the 2015 census [30, 31]; other areas were defined as rural areas. Additionally, the following information was used: sex; age; diagnosis year; diagnostic groups based on the International Classification of Childhood Cancer, third edition; clinical stage; and the type of school (pre [0–6 years old], elementary [6–12 years old], and junior/senior high school [12–18 years old]). The type of school was classified according to the age and month/year at diagnosis.

### Statistical methods

Count data are summarized as numbers, percentages, and, when necessary, 95% confidence intervals (CIs) calculated using the Clopper-Pearson estimation method. Continuous data that did not follow a normal distribution are summarized as numbers and medians. A Pareto plot was used to investigate the centralization of treatment across the diagnostic group [32, 33]. This plot draws a line symmetrical to the Lorenz curve [34], which represents the cumulative percentage of patients treated at each hospital, summed in descending order, starting with the highest number of patients treated at the hospital. If the line is 45°, it suggests that the same number of cases were treated at each facility (decentralized), and if more patients were treated at limited facilities, the curve moves to the upper left (centralized). A multivariable logistic model was performed to investigate the factors associated with events requiring car travel duration exceeding 1 h. The explanatory variables were sex, type of school, diagnostic group, type of hospital, urban/rural area, and region. The variance inflation factor was used to check the severity of multicollinearity.

The address geocoding was performed using the MAPPLE address-matching tool version 3.2.12.0630. The RAND function in SAS 9.4 was used to generate two random variables following the continuous uniform distribution to specify the degree and distance to move each coordinate; R version 4.1.0 and the Package “geosphere” were used to move the coordinates previously specified in SAS [35]. The NAVITIME Application Programming Interface service was used as a web route-planning web service for estimating travel. Moreover, SAS 9.4 was used for the analysis, including the REG procedure for the multivariable logistic model. The study was approved by the Ethical Review Board of Osaka University Hospital (Approval No. 19522(T2)-8). The initial data access date for the study population was December 21, 2022. The need for informed consent was waived because the study used existing anonymized data.

## Results

Of the 10,709 patients, 8,215 (76.7%) lived in urban areas and 2,494 (23.3%) lived in rural areas (Table 1). Patients aged 0–14 years accounted for 75.1% of all patients, and 38.6% were preschoolers. In the diagnostic group, leukemias (I) were the most common cancer type (31.6%), followed by CNS tumors (III) (18.8%) and lymphomas (II) (10.3%). Regarding the regions where patients lived, 36.7% lived in the Kanto-Koshin’etsu area (including the Tokyo metropolitan area), 17.1% lived in the Kinki area (including the Osaka metropolitan area), and 14.3% lived in the Tokai-Hokuriku area (including the Nagoya metropolitan area).

**Table 1:** Characteristics of patients.

Category	Urban area	Rural area	Total
N	8,215	2,494	10,709
0–14 years old, n (%)	6,224 (75.8)	1,818 (72.9)	8,042 (75.1)
Male, n (%)	4,429 (53.9)	1,342 (53.8)	5,771 (53.9)
Type of school, n (%)			
Preschool	3,246 (39.5)	885 (35.5)	4,131 (38.6)
Elementary school	1,882 (22.9)	594 (23.8)	2,476 (23.1)
Junior/senior high school	3,087 (37.6)	1,015 (40.7)	4,102 (38.3)
Diagnostic group, n (%)			
I. Leukemias	2,621 (31.9)	758 (30.4)	3,379 (31.6)
II. Lymphomas	839 (10.2)	263 (10.5)	1,102 (10.3)
III. Central nervous system tumors	1,535 (18.7)	473 (19.0)	2,008 (18.8)
IV. Neuroblastoma	400 (4.9)	106 (4.3)	506 (4.7)
V. Retinoblastoma	223 (2.7)	51 (2.0)	274 (2.6)
VI. Renal tumors	154 (1.9)	58 (2.3)	212 (2.0)
VII. Hepatic tumors	154 (1.9)	50 (2.0)	204 (1.9)
VIII. Malignant bone tumors	408 (5.0)	115 (4.6)	523 (4.9)
IX. Soft tissue sarcomas	457 (5.6)	104 (4.2)	561 (5.2)
X. Germ cell tumors	807 (9.8)	260 (10.4)	1,067 (10.0)
XI. Other malignant epithelial neoplasms	568 (6.9)	246 (9.9)	814 (7.6)
XII. Other and unspecified	49 (0.6)	10 (0.4)	59 (0.6)
Clinical stage^a^, n (%)			
Localized	2,966 (36.1)	899 (36.0)	3,865 (36.1)
Regional	1,239 (15.1)	443 (17.8)	1,682 (15.7)
Distant	1,033 (12.6)	294 (11.8)	1,327 (12.4)
Unknown	329 (4.0)	89 (3.6)	418 (3.9)
Region, n (%)			
Hokkaido	217 (2.6)	215 (8.6)	432 (4.0)
Tohoku	320 (3.9)	417 (16.7)	737 (6.9)
Kanto–Koshin’etsu	3,378 (41.1)	550 (22.1)	3,928 (36.7)
Tokai–Hokuriku	1,206 (14.7)	322 (12.9)	1,528 (14.3)
Kinki	1,658 (20.2)	168 (6.7)	1,826 (17.1)
Chugoku–Shikoku	566 (6.9)	393 (15.8)	959 (9.0)
Kyushu–Okinawa	870 (10.6)	429 (17.2)	1,299 (12.1)

n, number of patients; N, total number of patients.

^a^Not displayed for <10 patients whose cancer was in situ and 3,410–3,419 whose staging was not applicable, mainly for leukemias (I) and lymphomas (II).

**Fig 3:**
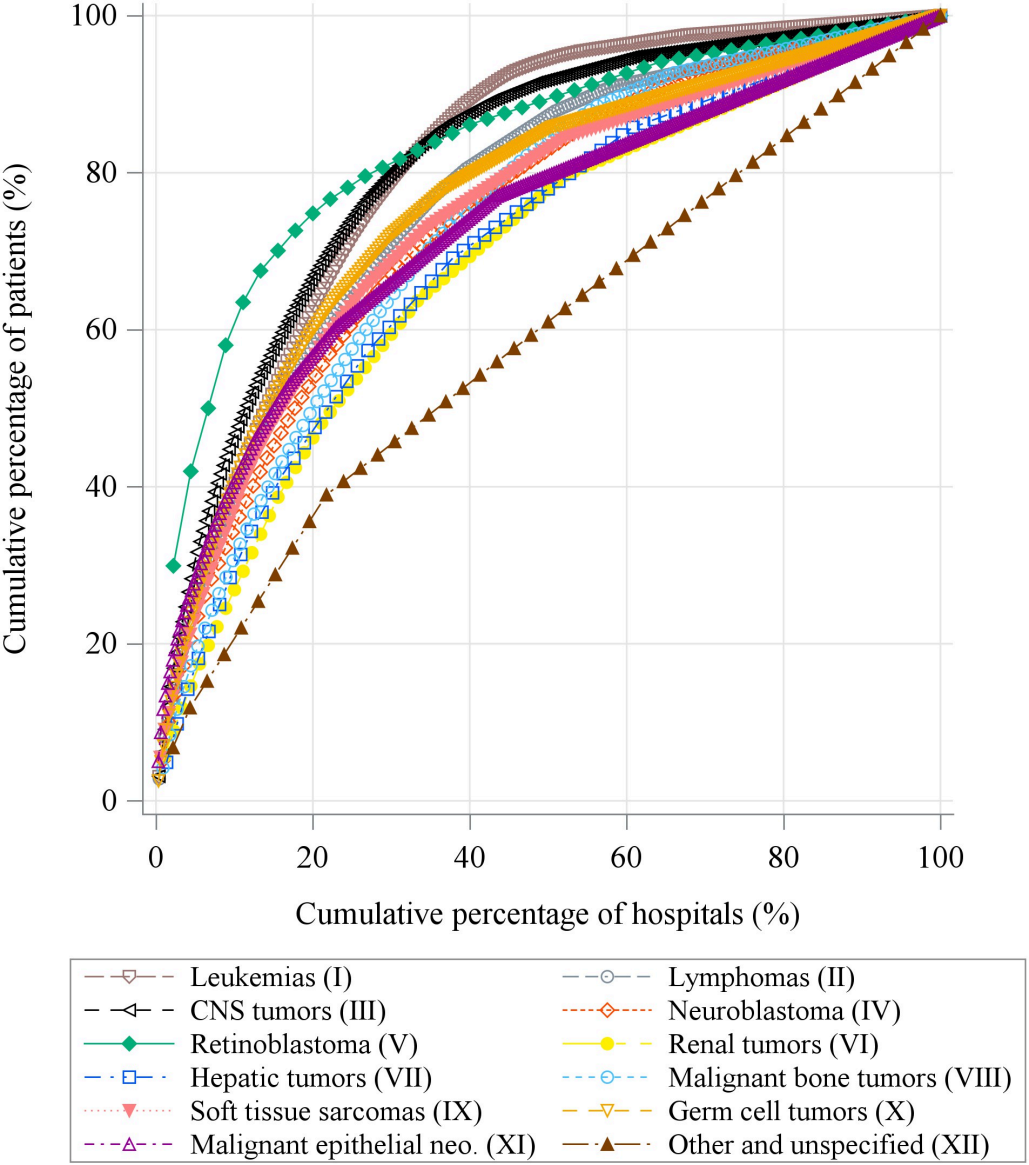
Pareto plot of the number of patients that were treated in each hospital by diagnostic group. CNS, central nervous system; Malignant epithelial neo., other malignant epithelial neoplasms. The x-axis presents the cumulative percentage of hospitals to the total number of hospitals that provided treatment by diagnosis group. The y-axis presents the cumulative percentage of patients treated at each hospital to the total number of patients by diagnosis group; the cumulative percentage of patients was calculated in the descending order of the number of patients treated in each hospital.

Overall, 4,804 patients received chemotherapy alone, and 2,357 patients received invasive treatment only (Table 2). For patients who received multiple types of treatments, the percentage of patients who received all treatments at the same hospital ranged from 80.0% to 88.6%. As presented in Table 3, 90.4% of patients were treated at designated hospitals, including 26.7% at childhood cancer-hub hospitals and 55.8% at other childhood cancer-specialized hospitals. A higher percentage of patients who lived in urban areas were treated at childhood cancer-hub hospitals (urban versus rural: 31.0% versus 12.7%), but the opposite trend was observed for other childhood cancer-specialized hospitals (urban versus rural: 51.3% versus 70.7%). Fig 3 presents the Pareto plot across diagnostic groups. The results suggest that the top five hospitals treated 63.5% of the patients with retinoblastoma (V) and that the top 20% of the hospitals treated 66.3% of the patients with CNS tumors (III). Conversely, only 35.6% of patients with unspecified cancers (XII), 45.6% of patients with renal tumors (VI), and 46.2% of patients with hepatic tumors (VII) were treated in the top 20% of the hospitals. Fewer than half (41.6%; 95% CI: 40.7–42.6) of the patients received at least one type of treatment within the secondary healthcare area in which they lived, indicating that the remaining patients received treatment outside their areas (Table 4). Regarding the type of treatment, 35.5% (95% CI: 33.3–37.8), 39.7% (95% CI: 38.6–40.8), and 41.6% (95% CI: 40.2–42.9) of patients who received radiotherapy, chemotherapy, and invasive treatment received treatment in the areas in which they lived, respectively. However, the percentages of patients receiving treatment in their own prefectures (any treatment: 86.5%) and regions (any treatment: 97.8%) were higher.

**Table 2:** Number of patients in various treatment combinations and percentage of patients who received all treatments in the same facility.

Treatment combinations	Urban area		Rural area		Total	
	n	Same facility (%)	n	Same facility (%)	n	Same facility (%)
Invasive treatment only	1,773	–	584	–	2,357	–
Radiotherapy only	114	–	22	–	136	–
Chemotherapy only	3,711	–	1,093	–	4,804	–
Invasive Trt + Radio	146	82.9	50	80.0	196	82.1
Invasive Trt + Chemo	1,339	88.6	404	87.1	1,743	88.3
Radio + Chemo	503	88.3	157	85.4	660	87.6
All	629	82.5	184	84.8	813	83.0

All, Invasive Treatment + Radiotherapy + Chemotherapy; Chemo, Chemotherapy; n, number of patients; Radio, Radiotherapy; Trt, Treatment.

**Table 3:** Number (percentage) of patients who received at least one type of treatment at designated hospitals.

Type of hospitals	Urban area	Rural area	Total
N	8,215	2,494	10,709
Childhood cancer hub-hospitals, n (%)	2,544 (31.0)	316 (12.7)	2,860 (26.7)
Other childhood cancer-specialized hospitals, n (%)	4,216 (51.3)	1,764 (70.7)	5,980 (55.8)
Other designated cancer hospitals, n (%)	639 (7.8)	201 (8.1)	840 (7.8)
Total (any of the above facilities), n (%)	7,399 (90.1)	2,281 (91.5)	9,680 (90.4)

n, number of patients; N, total number of patients.

**Table 4:** Percentage of patients who received their treatment within their residential area in the secondary healthcare area, the prefecture, and the region.

Treatment	n	Secondary healthcare area		Prefecture		Region	
		%	95% CI ^b^	%	95% CI ^b^	%	95% CI ^b^
Any Trt ^a^	10,709	41.6	40.7–42.6	86.5	85.8–87.1	97.8	97.5–98.1
Invasive Trt	5,109	41.6	40.2–42.9	83.8	82.8–84.8	96.8	96.3–97.3
Radiotherapy	1,805	35.5	33.3–37.8	80.0	78.1–81.8	95.1	94.0–96.1
Chemotherapy	8,020	39.7	38.6–40.8	86.8	86.0–87.5	98.1	97.8–98.4

CI, Confidence Interval; n, number of patients; Trt, Treatment.

The denominator represents the number of patients who received each treatment. The numerator is the number of patients whose residential areas were the same as the hospital’s location when examined at the regional level (secondary healthcare area, prefecture, or region), unless otherwise indicated.

^a^The numerator is the number of patients who received at least one treatment within the same residential area.

^b^95% Clopper–Pearson confidence interval.

Table 5 and S1 Table present the median estimated travel durations (hours), distances (km), and expenditures (dollars) for car travel; S2 Table presents those for transit travel. The estimated median travel burdens for patients were 0.62 h, 16.9 km, and 0.0 dollars of toll charges for car travel, respectively, and 1.08 h, 18.9 km, and 4.5 dollars of transit fees for transit travel, respectively. The estimated median travel burdens in rural areas were higher than that in urban areas (car travel: 0.57 h, 13.7 km, and $0.0 for urban areas vs 1.00 h, 48.5 km, and $3.9 for rural areas), and this finding was consistently observed across categories. Differences in the median car travel durations were observed between regions, diagnostic groups, and types of hospitals. In the multivariable logistic model, an event that exceeded 1 hour of estimated car travel duration was observed in 2,327 (21.7%) patients. A multicollinearity test revealed that the variance inflation factors were 1.1 at maximum. Rural areas were associated with a greater likelihood of estimated car travel duration exceeding 1 h (versus urban areas: adjusted odds ratio [OR]: 6.93; 95% CI: 6.17–7.78; Table 6). Additionally, when using leukemias (I) as a reference, retinoblastoma (V) (OR: 3.59; 95% CI: 2.71–4.77), malignant bone tumors (VIII) (OR: 1.94; 95% CI: 1.53–2.47), and hepatic tumors (VII) (OR: 1.45; 95% CI: 1.01–2.08) were associated with a greater likelihood of estimated car travel duration exceeding 1 h. Further, significant differences in the adjusted ORs were observed for the type of hospital and region.

**Table 5:** Number and median of estimated car travel durations (hours).

Category	Urban area		Rural area		Total	
	n	Med	n	Med	n	Med
All patients	8,215	0.57	2,494	1.00	10,709	0.62
Diagnostic group						
I. Leukemias	2,621	0.53	758	0.94	3,379	0.60
II. Lymphomas	839	0.57	263	1.00	1,102	0.62
III. Central nervous system tumors	1,535	0.60	473	1.10	2,008	0.67
IV. Neuroblastoma	400	0.59	106	1.11	506	0.65
V. Retinoblastoma	223	0.78	51	1.97	274	0.85
VI. Renal tumors	154	0.54	58	1.11	212	0.66
VII. Hepatic tumors	154	0.56	50	1.12	204	0.65
VIII. Malignant bone tumors	408	0.65	115	1.25	523	0.70
IX. Soft tissue sarcomas	457	0.53	104	0.93	561	0.58
X. Germ cell tumors	807	0.50	260	0.90	1,067	0.55
XI. Other malignant epithelial neoplasms	568	0.50	246	0.76	814	0.56
XII. Other and unspecified	49	0.43	10	0.60	59	0.43
Type of hospital						
Childhood cancer-hub hospitals	2,443	0.65	294	1.79	2,737	0.70
Other childhood cancer-specialized hospitals	4,215	0.55	1,743	0.95	5,958	0.63
Other designated cancer hospitals	681	0.42	218	0.48	899	0.43
Others	876	0.45	239	0.83	1,115	0.48
Region						
Hokkaido	217	0.45	215	1.88	432	0.72
Tohoku	320	0.50	417	1.17	737	0.83
Kanto–Koshin’etsu	3,378	0.60	550	0.91	3,928	0.63
Tokai–Hokuriku	1,206	0.57	322	0.85	1,528	0.62
Kinki	1,658	0.55	168	1.27	1,826	0.58
Chugoku–Shikoku	566	0.52	393	0.95	959	0.63
Kyushu–Okinawa	870	0.48	429	0.82	1,299	0.53

Med, Medians; n, number of patients.

**Table 6:** Odds ratios for the event that the estimated car travel duration exceeded 1 h.

Category	n	Event (%)	Crude		Adjusted	
			OR	95% CI	OR	95% CI
Sex						
Male (ref.)	5,771	1,226 (21.2)	1.00	-	1.00	-
Female	4,938	1,101 (22.3)	1.06	0.97–1.17	1.09	0.98–1.21
Type of school						
Preschool (ref.)	4,131	930 (22.5)	1.00	-	1.00	-
Elementary school	2,476	558 (22.5)	1.00	0.89–1.13	1.04	0.90–1.19
Junior/senior high school	4,102	839 (20.5)	0.89	0.80–0.98	0.98	0.86–1.13
Diagnostic group (%)						
I. Leukemias (ref.)	3,379	627 (18.6)	1.00	-	1.00	-
II. Lymphomas	1,102	214 (19.4)	1.06	0.89–1.26	1.06	0.88–1.28
III. Central nervous system tumors	2,008	508 (25.3)	1.49	1.30–1.70	1.54	1.33–1.79
IV. Neuroblastoma	506	120 (23.7)	1.36	1.09–1.70	1.39	1.08–1.77
V. Retinoblastoma	274	112 (40.9)	3.03	2.35–3.92	3.59	2.71–4.77
VI. Renal tumors	212	53 (25.0)	1.46	1.06–2.02	1.31	0.92–1.86
VII. Hepatic tumors	204	53 (26.0)	1.54	1.11–2.13	1.45	1.01–2.08
VIII. Malignant bone tumors	523	145 (27.7)	1.68	1.36–2.08	1.94	1.53–2.47
IX. Soft tissue sarcomas	561	117 (20.9)	1.16	0.93–1.44	1.36	1.07–1.73
X. Germ cell tumors	1,067	187 (17.5)	0.93	0.78–1.12	0.94	0.77–1.14
XI. Other malignant epithelial neoplasms	814	183 (22.5)	1.27	1.06–1.53	1.28	1.03–1.60
Type of hospital						
Others (ref.)	1,115	201 (18.0)	1.00	-	1.00	-
Childhood cancer-hub hospitals	2,737	646 (23.6)	1.40	1.18–1.68	1.91	1.56–2.33
Other childhood cancer-specialized hospital	5,958	1,365 (22.9)	1.35	1.15–1.59	1.12	0.93–1.35
Other designated cancer hospitals	899	115 (12.8)	0.67	0.52–0.85	0.57	0.43–0.74
Area						
Urban area (ref.)	8,215	1,100 (13.4)	1.00	-	1.00	-
Rural area	2,494	1,227 (49.2)	6.26	5.66–6.93	6.93	6.17–7.78
Region						
Kanto–Koshin’etsu (ref.)	3,928	729 (18.6)	1.00	-	1.00	-
Hokkaido	432	162 (37.5)	2.63	2.13–3.25	1.27	1.00–1.61
Tohoku	737	304 (41.2)	3.08	2.61–3.64	1.43	1.18–1.73
Tokai–Hokuriku	1,528	277 (18.1)	0.97	0.83–1.13	0.74	0.63–0.88
Kinki	1,826	282 (15.4)	0.80	0.69–0.93	0.80	0.68–0.94
Chugoku–Shikoku	959	266 (27.7)	1.68	1.43–1.98	0.97	0.81–1.16
Kyushu–Okinawa	1,299	307 (23.6)	1.36	1.17–1.58	0.91	0.77–1.07

CI, Confidence Interval; OR, Odds Ratio; ref, reference.

The analysis was based on a multivariable logistic model with the following explanatory effects: sex, type of school, diagnostic group, type of hospital, urban/rural area, and region. This analysis included patients with other and unspecified (XII) in the diagnostic group, though the results were not presented because the total number of patients with the event was less than 10.

## Discussion

This study investigated the latest situations in the travel burdens of children with cancer and their access to three types of treatment hospitals between 2016 and 2019 in Japan. Travel burden is considered a trade-off associated with the centralization of treatment; therefore, understanding the actual situation of the burden is important to provide optimal cancer care. Our findings suggest that most patients were treated in at least one specialized hospital designated for childhood cancer (Table 3), as in other high-income countries [2, 4]. Further, the median estimated burden of car travel was less than 1 h (0.62 h). This suggests that cancer control measures against childhood cancer in Japan have steadily promoted the centralization access to specialized hospitals while keeping the travel burden relatively manageable. This is remarkable compared to the situation before the establishment of the second term of the Basic Plan to Promote Cancer Control Programs in 2013 when numerous hospitals would treat only a handful of new cases per year and not provide up-to-date cancer care [19, 32, 33].

Two previous studies in Japan reported that most parents of patients were unwilling to travel one way for greater than 1 h [19], and a minority of inpatients aged 0–39 years (approximately 40%) had estimated car travel durations exceeding 45 min [20]. Similarly, in the United States, where 83.3% of patients travel less than 1 h to the nearest pediatric oncologist [36], cancer patients who traveled ≥1 h were a minority among patients aged 0–18 in Salt Lake City [10] and patients aged 0–39 in Pennsylvania [9]. In our study, 21.7% of cancer patients would have traveled over 1 h, which was lower than the percentages reported in these previous studies [9, 10, 20]. Moreover, urban and rural area-specific percentages were different (urban, 13.4%; rural, 49.2%; adjusted OR, 6.93), suggesting the existence of urban–rural disparities in travel burdens, as indicated in previous studies [9, 10, 20, 21]. This is because facilities that provide specialized treatment are generally located in urban areas, and patients and families are willing to travel to seek optimal care, if possible [15]. Nevertheless, across regions, the adjusted OR remained low, and the median travel duration was less than 1 h, even in the Hokkaido region, which is as large as the Republic of Austria. This suggests that even after the designation of specialized hospitals for childhood cancer, accessibility to these hospitals did not differ among all regions. However, additional consideration may be necessary in regions where greater burdens are observed, such as Hokkaido and Tohoku.

Some diagnostic groups exhibited significant differences in accessibility. Retinoblastoma (V) had the highest adjusted OR for travel >1 h (3.59), which was considered to involve the enhancement of treatment centralization, as revealed in Fig 3. Although the 5-year survival rate for retinoblastoma exceeds 90% [6], it accounts for only a small percentage of childhood cancer cases [37, 38]. Therefore, treatment may have been more centralized for this cancer type. By contrast, malignant bone tumors (VIII) and hepatic tumors (VII), which have similar rarities and lower survival rates than retinoblastoma (V) [6, 37, 38], had lower adjusted ORs for travel >1 h (1.94 and 1.45, respectively) and were treated in multiple hospitals. Nevertheless, the travel burden of retinoblastoma was considerably less than the averages of 421.8 km for European patients and 185.7 km for African patients [39]. Patients with CNS tumors (III), who have a 5-year survival rate of approximately 60% [6], had an adjusted OR for travel >1 h of 1.54, indicating the centralization trend. Considering these situations, the travel burden would increase more than that of retinoblastoma (V) if the treatment is centralized or the patient receives treatment at specialized hospitals. Further development of support systems—such as accommodation hotels and physical, psychological, and financial support—is necessary for patients and their families [15, 16, 19].

One strength of this study was that the travel routes were estimated as precisely as possible by utilizing the community-level location information and a web service of route planners developed by a Japanese vendor [40] under the consideration of personal information identification so that patients and hospitals are not identified by their community-level location information. For privacy reasons, the location data at the community level were randomly shifted 90 meters, or 0.09 km, at maximum from the geocoded location coordinates. Neither car nor transit travel would have been affected by estimation accuracy concerning distance and expenditures, while durations were slightly affected, considering the travel speeds per minute in each mode of transportation. However, while our results on expenditures might appear low even in rural areas, the actual round-trip costs would be more than twice that amount because they only considered one-way toll charges or transit fees per adult and not gasoline costs or transit fees for the number of people. As an additional strength, this web service usage enabled the reporting of results for public transportation transfers, which has rarely been performed in previous studies. A similar service by the Google Maps Platform Application Programming Interface was unavailable for searching transit travel in Japan at the time of this study [41]. Unlike existing Geographic Information System (GIS) software-based methods, this relatively novel method of using a route-planner web service is simple because it does not require GIS expertise [25] and is expected to be accurate based on the vendor’s proprietary machine-learning data and algorithms. However, due to the nature of using web services, this method requires address information to be exchanged over the network. Careful handling is required to avoid direct transmission of address information and to ensure confidentiality in preventing the identification of patients and hospitals.

Our study had seven main limitations. First, the estimated travel burdens were based on a 24-h average and did not consider traffic congestion or exact timetables, which failed to account for patients who needed to depart a day before the appointment. Hence, we summarized the burden using medians and a binary event. Second, a certain percentage of families would have relocated after the cancer diagnosis [10]. However, our study used patient addresses only at the time of diagnosis, which might result in an overestimation of the travel burdens for these patients. Third, our results indicated that the estimated values for transit travel were higher than those for car travel, but information regarding how patients actually traveled remained unavailable. Although patients and families may have preferred car travel to avoid crowds and people who were ill for patients in immunosuppressed states owing to the treatment side effects [42], some may have selected transit travel. Therefore, the actual median travel burden would have been higher, despite any patients having moved after diagnosis. Fourth, each hospital’s designation classification was determined by us based on the list of hospital names, and any overlap with the hospital’s designation period or the patient’s treatment period was not considered. Moreover, this study excluded patients with no information on whether they were treated, which resulted in a greater percentage of patients treated at designated hospitals, as indicated in Table 3. Fifth, expenditures in our study refer only to one-way toll charges or transit fees for one of the repeated travels to the hospital during long hospitalization or prolonged treatment and do not consider any other types of financial burdens, such as accommodation, relocation, arranging childcare for siblings, and leaving work for patients or caregivers [16]. Sixth, our data only covered a 4-year observation period and we did not conduct a time-series analysis to evaluate changes over time. Further studies will be necessary to corroborate the results based on other patient surveys and patient registries. Finally, we did not consider individual factors such as treatment or family characteristics that were not collected in the PBCR. Consequently, our results would remain prone to residual confounding from these unmeasured individual factors.

In conclusion, our population-based study in Japan indicated that treatment for childhood cancer is likely centralized, such that most patients were treated in at least one specialized hospital. However, only 21.7% of the patients were estimated to have spent greater than 1 h traveling one way by car. Cancer control measures against childhood cancer in Japan were considered to have steadily promoted the centralization of access to specialized hospitals while keeping the travel burden to a manageable level.

## Supporting information

S1 Table Numbers and medians of estimated travel durations (hours), distances (km), and expenditures (U.S. dollars) for car travel. Dist, Distances; Dur, Durations; Exp, Expenditures; n, number of patients. Expenditures are the sum of toll charges for a standard-sized car in U.S. dollars. All analyses used the durations, distances, and expenditures for the route with the shortest travel time among the routes to access each treatment hospital for each case, unless otherwise indicated. ^a^This analysis used durations, distances, and expenditures to access each treatment hospital.
(XLSX)

S2 Table Numbers and medians of estimated travel durations (hours), distances (km), and expenditures (U.S. dollars) for transit travel. Dist, Distances; Dur, Durations; Exp, Expenditures; n, number of patients. Expenditures are the sum of transit fees in adult price in U.S. dollars. All analyses used the durations, distances, and expenditures for the route with the shortest travel time among the routes to access each treatment hospital for each case, unless otherwise indicated. ^a^This analysis used durations, distances, and expenditures to access each treatment hospital.
(XLSX)
